# DNA Methylation of Five Core Circadian Genes Jointly Contributes to Glucose Metabolism: A Gene-Set Analysis in Monozygotic Twins

**DOI:** 10.3389/fgene.2019.00329

**Published:** 2019-04-12

**Authors:** Hao Peng, Yun Zhu, Jack Goldberg, Viola Vaccarino, Jinying Zhao

**Affiliations:** ^1^Department of Epidemiology, College of Public Health and Health Professions and College of Medicine, University of Florida, Gainesville, FL, United States; ^2^Department of Epidemiology, School of Public Health, University of Washington, Seattle, WA, United States; ^3^Department of Epidemiology, Rollins School of Public Health, Emory University, Atlanta, GA, United States

**Keywords:** circadian rhythm, DNA methylation, monozygotic twins, glucose metabolism, epigenetics

## Abstract

The timing of daily fluctuations in blood glucose is tightly controlled by the circadian rhythm. DNA methylation accompanies the circadian clock, and aberrant DNA methylation has been associated with circadian disruption and hyperglycemia. However, the precise role of circadian genes methylation in glucose metabolism is unknown. Using a gene-set approach in monozygotic (MZ) twin pairs, we examined the joint effect of 77 CpGs in five core circadian genes (*CLOCK*, *BMAL1*, *PER1*, *PER2*, *PER3*) on glucose-related traits in 138 middle-aged, male-male MZ twins (69 pairs). DNA methylation was quantified by bisulfite pyrosequencing. We first conducted matched twin pair analysis to examine the association of single CpG methylation with glucose metabolism. We then performed gene-based and gene-set analyses by the truncated product method to examine the combined effect of DNA methylation at multiple CpGs in a gene or all five circadian genes as a pathway on glucose metabolism. Of the 77 assayed CpGs, only one site was individually associated with insulin resistance at FDR < 0.05. However, the joint effect of DNA methylation in all five circadian genes together showed a significant association with glucose metabolism. Our results may unravel a biological mechanism through which circadian rhythm regulates blood glucose, and highlight the importance of testing the joint effect of multiple CpGs in epigenetic analysis.

## Introduction

Circadian rhythms regulate many vital biological processes including glucose metabolism and insulin secretion (Qian and Scheer, 2016). Dysregulation of the circadian system has been associated with metabolic disorders, e.g., hyperglycemia (Leproult et al., 2014), insulin resistance (Rao et al., 2015), obesity (Krueger et al., 2015), dyslipidemia (Chua et al., 2013), metabolic syndrome (Wang et al., 2014), and atherosclerosis (Jankowiak et al., 2016). However, the molecular mechanisms underlying the circadian control of glucose metabolism are incompletely understood.

The circadian clock is under the tight control of an endogenous transcription-translation feedback loop, which is regulated by many clock genes. Among these, the clock circadian regulator (*CLOCK*), brain and muscle aryl hydrocarbon receptor nuclear translocator-like 1 (*BMAL1*), and period circadian clock (*PER1/PER2/PER3*) represent the core circadian genes in peripheral or central tissues (Kalsbeek et al., 2014). Genetic polymorphisms in these clock genes may also be related to fasting glucose (Bouatia-Naji et al., 2009; Englund et al., 2009; Dupuis et al., 2010; Dashti et al., 2015), insulin resistance (Lyssenko et al., 2009; Dashti et al., 2015), obesity (Garaulet et al., 2014), and type 2 diabetes (Bouatia-Naji et al., 2009; Voight et al., 2010; Bonnefond et al., 2012; Gaulton et al., 2015). While these studies highlight the importance of genetic variants in the circadian control of glucose metabolism, DNA sequence variation only explains a small fraction of inter-individual variability in blood glucose. Epigenetic factors such as DNA methylation provide an interface between the fixed genome and dynamic environment, representing a mechanism through which our changing environment can affect glucose metabolism. In fact, dysregulation of DNA methylation has previously been related to various metabolic disorders such as diabetes (Chambers et al., 2015; Kulkarni et al., 2015; Al Muftah et al., 2016; Florath et al., 2016; Kriebel et al., 2016; Soriano-Tarraga et al., 2016), obesity (Demerath et al., 2015; Ding et al., 2015; Al Muftah et al., 2016; Kriebel et al., 2016), and insulin resistance (Dayeh et al., 2014; Hidalgo et al., 2014; Kriebel et al., 2016). Moreover, the circadian clock is epigenetically controlled and circadian disruption causes changes in DNA methylation of circadian genes (Zhu et al., 2011; Bhatti et al., 2015). For example, in a weight loss intervention trial including 60 normal weight and obese women, DNA methylation variation in promoter regions of the *CLOCK*, *BMAL1*, and *PER2* genes was associated with BMI, adiposity, weight loss, and metabolic syndrome (Milagro et al., 2012). An EWAS in subcutaneous adipose tissue from five pairs of MZ twins discordant for diabetes also revealed a significant enrichment of CpGs in genes involved in the circadian pathway, although no single CpG methylation was individually associated with diabetes (Ribel-Madsen et al., 2012). These results suggest that altered DNA methylation of circadian genes may play an important role in regulating glucose metabolism.

Previous epigenetic studies have largely focused on testing the individual effect of single CpG site, but as the effect of DNA methylation at a single CpG site could be very small, testing the individual effect of a single CpG site on a complex trait such as glucose metabolism may not be efficient. Statistical methods that can test the combined effects of multiple CpGs in multiple genes in a biological pathway may be a preferred approach in unraveling the genetic basis of complex traits.

Using a gene-set approach, the goal of this study is to examine the joint effect of DNA methylation at 77 CpG sites in five core circadian genes (*CLOCK*, *BMAL1*, *PER1*, *PER2*, *PER3*) on glucose metabolism in 69 MZ twin pairs participating in THS. Because MZ twin pairs share identical genetic materials and early life familial environment, a MZ co-twin control design rules out potential confounding by these factors and represents an ideal model for epigenetic analysis of human complex traits such as blood glucose.

## Materials and Methods

### Twin Pairs

The THS was designed to investigate the role of psychological, behavioral, and biological risk factors in subclinical cardiovascular disease in twins (Zhao et al., 2012b). Briefly, the study enrolled 180 middle-aged, male-male twin pairs who were born between 1946 and 1956 from VETR. All twins were free of overt cardiovascular disease at the time of enrollment and were examined in pairs at the Emory University General Clinical Research Center between 2002 and 2010. Venous blood samples were drawn by trained nurse in the morning (6:30∼7:00) after an overnight fast and stored at -80°C until laboratory tests. Zygosity was determined by DNA typing. All twins provided written informed consent. The protocols for both the THS and the current study were approved by the Emory University Institutional Review Board. Of the 84 MZ twin pairs with available DNA sample and clinical data for both members of a twin pair, we removed 15 twin pairs who were either concordant or discordant on overt type 2 diabetes in order to eliminate the potential impact of hypoglycemic drugs on the association between DNA methylation and glucose metabolism. The final data analysis included 69 complete MZ pairs.

### DNA Methylation Measurement

DNA methylation levels in the promoter regions of five key circadian genes including *CLOCK*, *BAML1*, *PER1*, *PER2*, and *PER3* were quantified by bisulfite pyrosequencing using genomic DNA isolated from peripheral blood leukocytes, as previously described (Zhao et al., 2013). In brief, genomic DNA was bisulfite treated using the EZ DNA Methylation Kit (Zymo Research, Inc., CA, United States) according to the manufacturer’s protocol, which converts un-methylated cytosines into uracil, and leaves methylated cytosines unchanged. Pyrosequencing was done on the PSQ96 HS System (Pyrosequencing, Qiagen). The targeted CpG sites in each gene were chosen based on: (a) CpG probes in the Illumina 450K array and RRBS databases; (b) regulatory elements such as promoter, enhancer, transcriptional binding sites, and (c) genomic sequences from UCSC genome browser. Genomic coordinates in Genome Reference Consortium Human Build 37 (GRCh37). The genomic coordinates and targeted sequences for pyrosequencing were shown in Supplementary Table S1 and schematically illustrated in Supplementary Figure S1. Methylation level at each CpG site was calculated as the percentage of the methylated alleles over the sum of methylated and un-methylated alleles. For quality control, each experiment included non-CpG cytosines as internal control to verify completion of sodium bisulfite DNA conversion. Un-methylated and methylated DNAs were also included as controls in each run. Pyrosequencing assay was run in duplicates, with a high correlation (≥99.8%) of two runs for a same sample.

### Measurement of Glucose Metabolism

Levels of blood glucose, insulin, and HbA1c in fasting plasma were measured on the Beckman CX7 chemistry auto analyzer (Beckman Coulter Diagnostics). Insulin resistance was assessed by the homeostasis model assessment (HOMA-IR) based on fasting glucose and fasting insulin (Duncan et al., 1995). All biochemical assays for each twin pair were processed in the same analytical run.

### Measurement of Risk Factors

Body weight (kg) and height (cm) were measured when twin participants wore light clothes and no shoes by trained research staff. BMI was calculated by dividing weight in kilograms by the square of height in meters (kg/m^2^). Systolic (SBP) and DBP were measured by a mercury sphygmomanometer on the right arm in sitting position after at least 10 min of rest. Two separate blood pressure measurements, taken 5 min apart, were averaged for analysis. Cigarette smoking was classified into current smoker (any number of cigarettes) versus never or past smoker. Pack-years of smoking were calculated as the number of packs of cigarettes smoked per day times the number of years smoked. Information on alcoholic (wine, beer, or cocktail) beverages consumed within a typical week was obtained and alcohol consumption (g/week) was estimated as previously described (Zhao et al., 2012a). Physical activity was assessed by means of the Baecke physical activity score, which summarizes activity related to work, sports, and leisure (Richardson et al., 1995). HDL-c and LDL-c were measured with homogeneous assays (Equal Diagnostics, Exton, Pennsylvania). Triglycerides were determined by enzymatic methods (Beckman Coulter Diagnostics, Fullerton, CA, United States). Depressive symptoms were measured with BDI-II (Beck et al., 1996).

### Statistical Analysis

Monozygotic twin pairs share 100% of their genes and early life environment including uterine environment, all of which could influence the epigenome and metabolic risk (Gluckman et al., 2009). To eliminate the potential confounding by shared gene and environment, we conducted a co-twin control matched pair analysis using within-pair differences (Zhao et al., 2015). To achieve this, we first calculated the intra-pair difference in DNA methylation, defined as the difference in methylation level of each CpG site between members of a twin pair. The intra-pair differences in HOMA-IR, fasting glucose, and HbA1c as well as continuous covariates including cigarette smoking (pack-year), alcohol consumption (g/week), physical activity level, BMI, lipids (HDL-c, LDL-c), SBP, and depressive symptoms (BDI-II score) were similarly calculated. To examine whether DNA methylation of circadian genes influences glucose metabolism, we simultaneously conducted single CpG analysis, gene-based and gene-set association analyses. All statistical analyses were performed using SAS statistical software (version 9.4, Cary, NC, United States).

### Single CpG Association Analysis

To examine the association of DNA methylation at each single CpG site with glucose metabolism, we constructed a linear regression model (Carlin et al., 2005), in which intra-pair difference in a glucose trait (e.g., HOMA-IR, fasting glucose, or HbA1c) was the dependent variable and intra-pair difference in DNA methylation level at each CpG site was the independent variable, adjusting for twin age and intra-pair differences in covariates listed above. Multiple testing was controlled by adjusting for the total number of CpG sites using FDR, and a FDR-adjusted *P*-value (i.e., *q*-value) less than 0.05 was considered statistically significant.

### Gene-Based and Gene-Set Association Analyses

To examine the joint effect of DNA methylation at multiple CpG sites in a circadian gene or all five circadian genes as a pathway on glucose metabolism, we employed a wTPM as previously described (Zaykin et al., 2002). This method tests the joint effects of multiple CpG sites in a gene or multiple genes in a pathway by combining *P*-values of all CpGs in a chosen gene or all genes in a selected pathway. The effect size of DNA methylation at each CpG site (i.e., regression coefficient) was included as weight in the TPM statistic. Multiple testing for gene-based analysis was controlled by adjusting for total number of genes. This method has been evaluated extensively by simulation studies (Sheng and Yang, 2013), and our group has used this approach to test the joint associations of genetic variants in the smoking gene pathway with diabetes (Yang et al., 2012), subclinical atherosclerosis (Yang et al., 2013), kidney function (Zhu et al., 2014a), and obesity (Zhu et al., 2014b). Here we extended this approach to epigenetic analysis.

## Results

The current analysis included 69 male-male MZ twin pairs (mean age 55 years). All twins are European Caucasians and were free of overt diabetes and cardiovascular disease at the time of blood draw. Clinical characteristics of the twins are presented in Table 1.

**Table 1:** Characteristics of monozygotic twin pairs (*N* = 69 pairs).

Characteristics	Mean ± SD
No. of twins	138
Age (years)	54.8 ± 2.9
Cigarette smoking (pack-year)	22.3 ± 23.8
Alcohol consumption (g/week)	12.5 ± 38.0
Physical activity level	7.5 ± 1.7
BDI-II score	6.0 ± 6.6
Body mass index (kg/m^2^)	28.9 ± 4.9
HOMA-IR	1.87 ± 1.34
Fasting plasma glucose (mg/dL)	97.2 ± 11.2
HbA1c (%)	5.5 ± 0.5
Systolic blood pressure (mmHg)	129.5 ± 15.4
Diastolic blood pressure (mmHg)	81.6 ± 10.9
High-density lipoprotein cholesterol (mg/dL)	38.5 ± 10.9
Low-density lipoprotein cholesterol (mg/dL)	123.7 ± 35.0
Total cholesterol (mg/dL)	186.9 ± 35.9
Triglycerides (mg/dL)	169.3 ± 96.5


*BDI-II, the Beck Depression Inventory-II; HOMA-IR, the homeostasis model assessment of insulin resistance; HbA1c, hemoglobin A1c*.

### Single CpG Association Analysis

Of the 77 assayed CpG sites in the promoter regions of the five circadian genes, methylation variation at 6 CpG sites (3 in *CLOCK*, 2 in *BMAL1*, 1 in *PER2*) was significantly associated with insulin resistance, methylation at 8 CpG sites (4 in *CLOCK*, 2 in *BMAL1*, 2 in *PER2*) was associated with fasting glucose, and methylation at 3 CpG sites (2 in *BMAL1*, 1 in *PER2*) was associated with HbA1c (all *P* < 0.05), after correction for covariates. After further adjusting for multiple testing, the association between DNA methylation at only 1 CpG site in the *BMAL1* gene remained to be statistically significant in relation to insulin resistance at *q* < 0.05. Table 2 presents the results of single CpG association analysis. Supplementary Table S2 shows the mean intra-pair differences in DNA methylation at each of the assayed CpG sites.

**Table 2:** Association of single CpG methylation in five core circadian genes with glucose metabolism (raw *P* ≤ 0.1).

Genomic position (GRCh37)	Relative to TSS (bp)	Methylation level (%, Mean ± SD)	HOMA-IR		Fating glucose		HbA1c	
			β (SE)	*P*^∗^	β (SE)	*P*^∗^	β (SE)	*P*^∗^
***CLOCK* (Chr4)**
56413146	158	0.78 ± 1.11	0.160 (0.101)	0.115	2.135 (1.144)	0.068	0.031 (0.048)	0.516
56413153	151	1.06 ± 1.19	0.165 (0.092)	0.073	1.894 (1.042)	0.076	0.044 (0.044)	0.329
56413164	140	1.90 ± 1.35	0.067 (0.092)	0.465	2.207 (1.003)	0.033	0.058 (0.042)	0.169
56413207	97	1.15 ± 1.03	0.204 (0.102)	0.044	1.433 (1.167)	0.226	0.038 (0.049)	0.444
56413229	75	2.20 ± 1.04	0.292 (0.100)	0.003	2.863 (1.130)	0.015	0.087 (0.047)	0.073
56413260	44	0.65 ± 1.18	0.137 (0.079)	0.085	0.760 (0.910)	0.408	-0.004 (0.037)	0.921
56413277	27	0.66 ± 1.00	0.299 (0.116)	0.010	3.213 (1.328)	0.020	0.038 (0.057)	0.506
56413308	-4	1.36 ± 1.24	0.129 (0.086)	0.132	2.326 (0.970)	0.021	0.051 (0.041)	0.216
56413348	-44	0.74 ± 1.06	0.153 (0.108)	0.158	2.300 (1.193)	0.060	0.079 (0.049)	0.118
***BMAL1* (Chr11)**
13298892	-453	0.85 ± 1.11	0.530 (0.179)	**0.003**	5.586 (2.097)	0.011	0.228 (0.088)	0.013
13298896	-449	0.38 ± 0.65	0.272 (0.171)	0.110	3.308 (1.988)	0.103	0.149 (0.080)	0.068
13298902	-443	1.24 ± 0.99	0.169 (0.218)	0.439	3.999 (2.441)	0.108	0.230 (0.097)	0.022
13298923	-422	0.34 ± 0.72	-0.389 (0.222)	0.080	-1.291 (2.565)	0.617	-0.105 (0.112)	0.353
13298932	-413	1.59 ± 0.93	0.074 (0.106)	0.484	2.169 (1.160)	0.068	0.065 (0.048)	0.182
13298934	-411	0.74 ± 0.89	0.254 (0.122)	0.037	2.795 (1.404)	0.053	0.114 (0.058)	0.054
13298975	-370	1.19 ± 0.98	0.133 (0.102)	0.193	2.286 (1.135)	0.050	0.026 (0.048)	0.594
13299014	-331	2.56 ± 1.48	0.046 (0.068)	0.501	1.426 (0.739)	0.060	0.039 (0.031)	0.207
13299033	-312	2.03 ± 1.21	0.137 (0.078)	0.080	1.917 (0.900)	0.039	0.059 (0.037)	0.120
***PER1* (Chr17)**
8055788	-36	1.29 ± 1.03	0.155 (0.138)	0.261	2.873 (1.519)	0.065	0.030 (0.065)	0.643
8055794	-42	1.82 ± 0.95	0.186 (0.134)	0.163	2.935 (1.493)	0.055	0.055 (0.062)	0.380
8055831	-79	1.16 ± 0.96	-0.219 (0.122)	0.073	-0.597 (1.409)	0.674	0.027 (0.058)	0.646
8055883	-131	0.93 ± 1.08	-0.186 (0.104)	0.074	-0.356 (1.209)	0.770	0.021 (0.049)	0.674
***PER2* (Chr2)**
239197609	-361	1.19 ± 0.79	0.123 (0.118)	0.299	2.315 (1.360)	0.096	0.068 (0.056)	0.235
239197627	-379	0.37 ± 0.40	0.494 (0.253)	0.051	4.415 (2.868)	0.131	0.164 (0.118)	0.173
239197634	-386	0.41 ± 0.51	0.509 (0.213)	0.017	6.068 (2.382)	0.014	0.127 (0.102)	0.217
239197649	-401	0.73 ± 0.43	0.368 (0.283)	0.193	7.025 (3.202)	0.033	0.316 (0.132)	0.021
***PER3* (Chr1)**
7844662	-53	41.73 ± 5.94	-0.041 (0.033)	0.222	-0.207 (0.379)	0.589	-0.030 (0.015)	0.051
7844735	20	45.60 ± 14.09	-0.014 (0.008)	0.068	0.074 (0.091)	0.418	0.001 (0.004)	0.86
7844743	28	44.36 ± 14.96	0.015 (0.009)	0.091	-0.096 (0.103)	0.357	0.000 (0.004)	0.954


*^∗^Adjusted for twin age and intra-pair differences in pack-year, alcohol consumption, physical activity, body mass index, systolic blood pressure, low and high-density lipoprotein cholesterol, and depressive symptoms. *P*-values in bold indicate significant association at *q* < 0.05 after adjusting for multiple testing. TSS, transcription start site*.

### Gene-Based and Gene-Set Association Analyses

At the level of *q* < 0.05, gene-based analysis detected significant associations of DNA methylation in four circadian genes (*CLOCK*, *BMAL1*, *PER1*, and *PER2*) with insulin resistance, fasting glucose, or HbA1c. Gene-set analysis revealed that DNA methylation in all five circadian genes as a pathway jointly contributed to insulin resistance (*P* = 0.0001), fasting glucose (*P* = 0.0001), and HbA1c (*P* = 0.0005). Results for gene-based and gene-set association analyses are shown in Table 3.

**Table 3:** Gene-based and gene-set associations of DNA methylation in five core circadian genes with glucose metabolism.

Circadian genes	HOMA-IR		Fasting glucose		HbA1c	
	Raw *P*	*q*-Value	Raw *P*	*q*-Value	Raw *P*	*q*-Value
**Gene-based association analysis**
*CLOCK*	0.0003	0.0008	0.0003	0.0007	0.4556	0.4556
*BMAL1*	0.0012	0.0024	0.0001	0.0007	0.0003	0.0011
*PER1*	0.0407	0.0543	0.0255	0.0357	0.3364	0.4052
*PER2*	0.0054	0.0086	0.0014	0.0025	0.0404	0.0943
*PER3*	0.4474	0.4474	0.3730	0.373	0.1048	0.1834
**Gene-set association analysis**
Circadian pathway	0.0001		0.0001		0.0005	


## Discussion

Using a gene-family approach, we demonstrated that altered DNA methylation in five circadian genes jointly contributed to glucose metabolism in a well-matched MZ twin sample. Our results may unravel a molecular mechanism through which circadian rhythms affect glucose metabolism, and suggest that simultaneously testing the joint effects of multiple CpG sites in a gene or multiple genes in a pathway is a powerful approach in epigenetic analysis of human complex traits.

Several aspects of our results merit discussion. First, in line with previous studies,(Shah et al., 2015) we also found that individual effect of a single CpG methylation on glucose metabolism is small. Of the 77 CpG sites assayed in our sample, DNA methylation at 10 CpG was nominally associated with one or more glucose traits (raw *P* < 0.05), with only one site survived multiple testing (*q* < 0.05). However, the combined effects of multiple CpG sites could be large. For example, in our single CpG analysis, DNA methylation at two out of the nine assayed CpG sites in the *PER2* gene showed marginal association with fasting glucose, but both disappeared after multiple testing correction. In contrast, gene-based test revealed a significant association of all nine CpG sites in this gene with fasting glucose. Similarly, of the 30 CpG sites measured in the promoter region of the *CLOCK* gene, only 4 CpG sites showed nominal individual associations with either blood glucose or insulin resistance, but gene-based approach detected a significant association of all 30 CpG sites in this gene with both blood glucose and insulin resistance. This highlights the importance of testing the joint epigenetic effect of multiple CpGs on human complex traits. Second, a complex trait such as glucose metabolism is most likely regulated by many genes in one or more biological pathways. Testing the individual effect of each individual gene may not be efficient and also does not reflect the true biology. We thus examined the combined effect of five core circadian genes as a pathway on glucose metabolism. It shows that, although not all genes showed significant individual association, the circadian pathway as a whole was significantly associated with glucose measures. Together, our findings suggest that testing the joint epigenetic effects of multiple CpG sites in a gene or a pathway represents a preferred approach for epigenetic analysis of human complex traits. Finally, although several previous studies reported associations of individual CpG methylation in the *CLOCK*, *BMAL1*, and *PER2* genes with glucose-related traits, e.g., insulin resistance, obesity (Milagro et al., 2012), no study has systematically tested the joint effect of multiple CpGs in a candidate gene or the circadian pathway on glucose metabolism.

Our study has several limitations. First, given that DNA methylation is tissue- or cell-type specific, it is unclear whether or to what extent the results derived from peripheral blood could reflect methylation changes in the target organs of glucose metabolism, e.g., pancreas, liver, fat and muscle. However, accumulating evidence indicated that epimutations may not be limited to the affected tissue but could also be detected in peripheral blood (Menke and Binder, 2014). Second, as the original THS was designed to examine the role of psychosocial factors in subclinical atherosclerosis, the study included multiple twin pairs discordant on major depression (37 MZ discordant pairs were included in the current analysis). It is unclear whether and how such bias will affect our results. However, we believe this should not be a major concern because we controlled for depressive symptoms in all statistical analyses. Third, as all observational studies, we cannot establish the causal role of DNA methylation in glucose metabolism. Moreover, as all studies using MZ twins, the sample size of our study is small. We cannot rule out the possibility of false positive or false negative findings, and thus our results need further replication. Fourth, the present study is a secondary data analysis based on genes assayed in a previous candidate gene-based study that did not include all the circadian genes (e.g., *CRY2*). Fifth, there is a time interval (about 5–9 years) between the biochemical and methylation assays. However, we believe that this should not be major concern because it has been shown that methylation profiles are stable in DNA or blood samples archived for up to 20 years under appropriate storage conditions (Li et al., 2018). Finally, as all twins included in the current analysis are European Caucasians, the generalizability of our results to other racial/ethnic groups is uncertain.

Nonetheless, our study has several strengths. First, as MZ twin pairs share identical genotypes, age, and sex, as well as many other socioenvironmental factors including in utero and familial environment, the use of a MZ co-twin control design minimizes or eliminates potential confounding by these factors. Moreover, our analyses also controlled for many lifestyles and metabolic factors. Second, we used innovative statistical approaches to test the combined effects of multiple CpG sites in a gene or multiple genes in the circadian pathway in well-matched MZ twins. To the best of our knowledge, we are the first to systematically test the joint epigenetic effect of multiple CpGs in the circadian pathway on glucose traits in MZ twins. Our results provide initial evidence that altered DNA methylation of key circadian genes may play an important role in glucose metabolism.

In summary, our results demonstrated that DNA methylation of the five core circadian genes jointly contributed to blood glucose metabolism. These findings also highlight the importance of testing the effect of DNA methylation jointly rather than separately on human complex traits in epigenetic analysis.

## Author Contributions

HP and YZ conducted the analysis. HP drafted the manuscript. JG and VV provided the critical comments. JZ designed the study and finalized the manuscript. All authors read and approved the final manuscript.

## Conflict of Interest Statement

The authors declare that the research was conducted in the absence of any commercial or financial relationships that could be construed as a potential conflict of interest.

## Abbreviations

BDI-II: the Beck Depression Inventory-II
BMI: body mass index
DBP: diastolic blood pressures
EWAS: epigenome-wide association study
FDR: false discovery rate
HbA1c: hemoglobin A1c
HDL-c: high-density lipoprotein cholesterol
HOMA-IR: the homeostasis model assessment of insulin resistance
LDL-c: low-density lipoprotein cholesterol
MZ: monozygotic
SBP: systolic blood pressure
THS: the Twins Heart Study
VETR: the Vietnam Era Twin Registry
wTPM: weighted truncated product method.
