# Supplementary material for: DNA Methylation of Five Core Circadian Genes Jointly Contributes to Glucose Metabolism: A Gene-Set Analysis in Monozygotic Twins
Source: Front Genet. 2019 Apr 12;10:329. doi: 10.3389/fgene.2019.00329 (PMC6473046; doi:10.3389/fgene.2019.00329)
Supplement: Supplementary file 1 [file Data_Sheet_1.pdf]

Online Supplementary data

Table S1. Genomic coordinates and targeted sequences for pyrosequencing

Gene Name	Genomic Location (GRCh37, bp)	Distance to TSS (bp)	# of CpG	Genomic Target Sequence	Bisulfite Converted Target Sequence
BMAL1	Chr11:13255468-13255510	-453 to -411	8	CCCGGGCGACCCCGAGGAGCGCGGCTTGGG CACCGCAGTGGCCGCGGCTAGTGGG	TTYGGGYGATTTYGAGGAGYGYGGTTTGG GTATYGTAGTGGTYGYGGTTAGTGGG
BMAL1	Chr11:13255551-13255609	-370 to -312	9	AGGGGAAAGGGAGAGGGCAGGGGCGAGGA ACCCAGGGAGCGCG/TCGCGGATTGGTCCTC TCCTCGGGGCGTGCG/ACTCCTGTGCGCCAA ATGA	AGGGGAAAGGGAGAGGGTAGGGGYGAGG AATTTAGGGAGYGTG/TYGYGGATTGGTTT TTTTTTYGGGGYGTGTG/ATTTTTGTGYGTT AAATGA
CLOCK	Chr4:56413357-56413261	-52 to 45	15	CCTAAGGCTAGCGCTCTGCCGGCCGGCCGCG CCCTCCACCCGACCAGGCCCCGCCTCCCCGG CTTTCTAGTAACCGGCGCCGTTCCCGGCCGG GGCAGGGAACGGTGCGCCTGCAGAGCCAGA	TTTAAGGTTAGYGTTTTGTYGGTYGGTYG YGTTTTTTATTYGATTAGGTTTYGTTTTTTY GGTTTTTTAGTAATYGGYGTYGTTTTYGGT YGGGGTAGGGAAYGGTGYGTTTGTAGAGT TAGA
CLOCK	Chr4:56413261-56413147	64 to 159	15	TTCGGCCCAAGGGGCGCGGGAGTCTCTTCGG GCGTCCGGGATCCCCTGGCGCGGCTCCGTGC TGCCTAACGGGGCAAGTCGCATGCGCACCG AGCCGCGCTGGGGAAA	TTYGGTTTAAGGGGYGYGGGAGTTTTTTY GGGYGTTYGGGATTTTTTGGYGYGGTTTY GTGTTGTTTAAYGGGGTAAGTYGTATGYG TATYGAGTYGYGTTGGGGAAA
PER1	Chr17:8055893-8055885	-140 to -132	3	AGGTCCACGTGCGCCCGTGTGTGTGACACAG CCCT	AGGTTTAYGTGYGTTYGTGTGTGTGATAT AGTTTT
PER1	Chr17:8055835-8055786	-82 to -33	7	GACCACTAGCCAATCAGGCGCCGGGAAGAG ATCCCCAGCCAATCGGGGGCGGGGCCTGCG GCTCCGTCGGCAAGAGG	GATTATTAGTTAATTAGGYGTYGGGAAGA GATTTTTAGTTAATYGGGGGYGGGGTTTG YGGTTTYGTYGGTAAGAGG
PER2	Chr2:239197651-239197608	-401 to -358	9	AGGACGACGGGTAGCACGAACGCGCCGCGT CTCCATTGAGGAACCGACGAGGTGAA	AGGAYGAYGGGTAGTAYGAAYGYGTYGY GTTTTTATTGAGGAATYGAYGAGGTGAA
PER3	Chr1:7844624-7844664	-90 to -50	9	CCGCGCGGTCGGGGCGCCCCAGCCAGCCGG GCCGCTGGCGTCGGACTGTCTG	TYGYGYGGTYGGGGYGTTTTAGTTAGTYG GGTYGTTGGYGTYGGATTGTTTG
PER3	Chr1:7844716-7844742	3 to 29	4	GCCGGAGTCCTGAAAGTCGAGCGAGCTCCG GGTTTTGAAAA	TYGGAGTTTTGAAAGTYGAGYGAGTTTYG GGTTTTGAAAA

Table S2. Mean intra-pair differences in each CpG site

Genomic position (GRCh37)	mean Δ	Genomic position (GRCh37)	mean Δ	Genomic position (GRCh37)	mean Δ	Genomic position (GRCh37)	mean Δ
***CLOCK* (Chr4)**		56,413,291	-0.277	13,298,995	-0.192	239,197,633	-0.036
56,413,147	0.128	56,413,294	-0.245	13,298,997	-0.339	239,197,635	-0.023
56,413,149	-0.142	56,413,309	-0.282	13,299,014	-0.170	239,197,639	0.018
56,413,154	0.327	56,413,317	-0.452	13,299,019	-0.007	239,197,648	-0.143
56,413,159	0.089	56,413,328	0.294	13,299,033	-0.257	239,197,651	-0.226
56,413,165	-0.141	56,413,339	0.093	***PER1*(Chr17)**		***PER3* (Chr1)**	
56,413,175	0.036	56,413,341	-0.004	8,055,786	-0.274	7,844,624	0.293
56,413,187	-0.204	56,413,345	-0.168	8,055,789	-0.425	7,844,626	0.363
56,413,193	0.112	56,413,349	0.175	8,055,795	-0.272	7,844,628	1.079
56,413,195	0.107	56,413,357	0.069	8,055,804	-0.091	7,844,632	-0.243
56,413,208	-0.234	***BMAL1* (Chr11)**		8,055,810	0.078	7,784,577	
56,413,212	0.021	13,298,892	-0.063	8,055,832	-0.107	7,844,650	0.884
56,413,216	0.029	13,298,896	0.111	8,055,835	-0.058	7,844,655	0.155
56,413,228	-0.086	13,298,902	-0.143	8,055,885	-0.316	7,844,661	0.462
56,413,230	0.166	13,298,909	-0.177	8,055,889	-0.191	7,844,664	-0.308
56,413,242	-0.232	13,298,911	-0.046	8,055,893	-0.092	7,844,716	-1.087
56,413,261	0.233	13,298,923	-0.215	***PER2* (Chr2)**		7,784,671	
56,413,266	0.339	13,298,932	-0.112	239,197,608	0.003	7,844,735	-0.861
56,413,278	0.218	13,298,934	0.075	239,197,611	-0.033	7,844,743	0.871
56,413,282	-0.259	13,298,975	0.104	239,197,628	0.003		
56,413,288	0.046	13,298,991	-0.201	239,197,630	-0.006		

Figure S1. Assayed CpG sites in the five circadian-related genes

***CLOCK* gene**

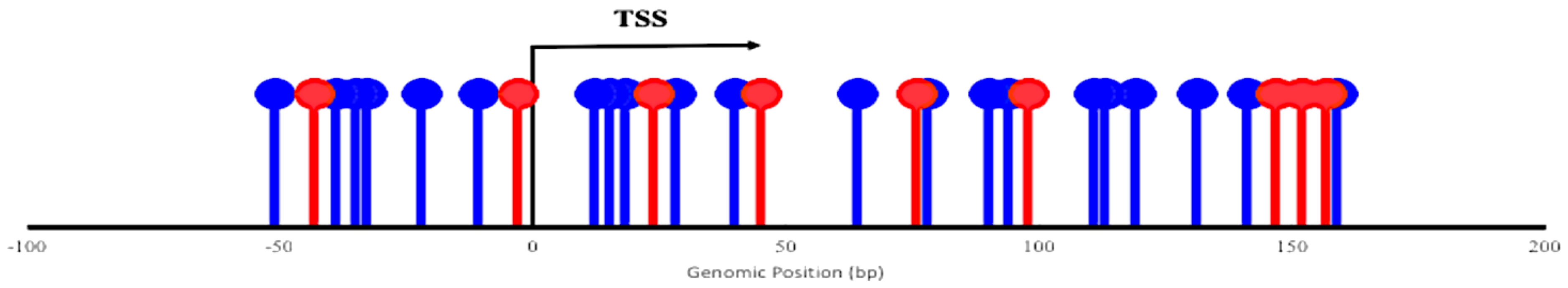

Figure S1A. A schematic illustration of the assayed CpG sites in the *CLOCK* gene in the study. A total of 30 CpG sites were assayed in the *CLOCK* gene. Red represents the CpG sites associated with glucose metabolism (raw P ≤ 0.1). TSS: transcription start site.

***BMAL1* gene**

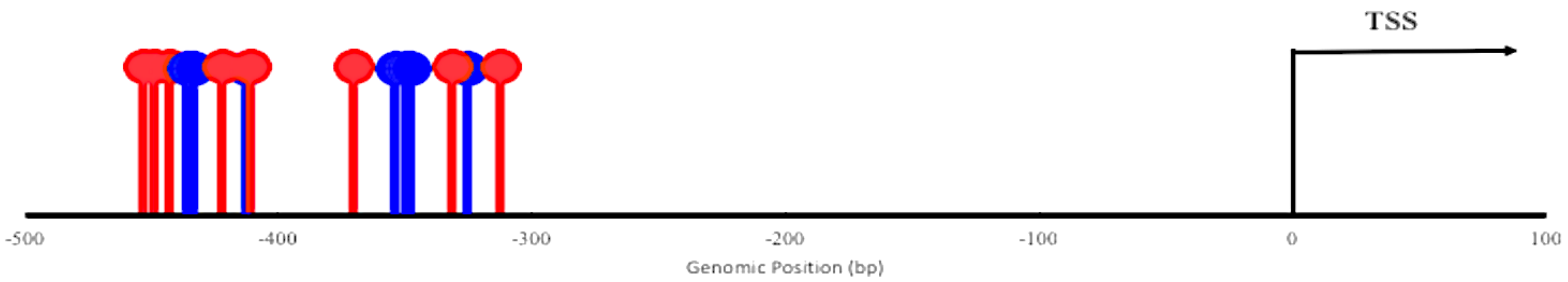

Figure S1B. A schematic illustration of the assayed CpG sites in the *BMAL1* gene in the study. A total of 17 CpG sites were assayed in the *BMAL1* gene. Red represents the CpG sites associated with glucose metabolism (raw P ≤ 0.1). TSS: transcription start site.

***PER1* gene**

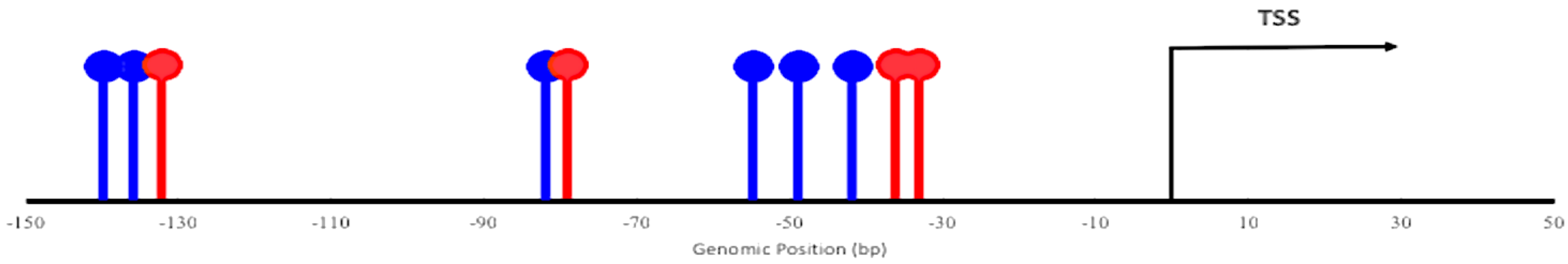

Figure S1C. A schematic illustration of the assayed CpG sites in the *PER1* gene in the study. A total of 10 CpG sites were assayed in the *PER1* gene. Red represents the CpG sites associated with glucose metabolism (raw P ≤ 0.1). TSS: transcription start site.

***PER2* gene**

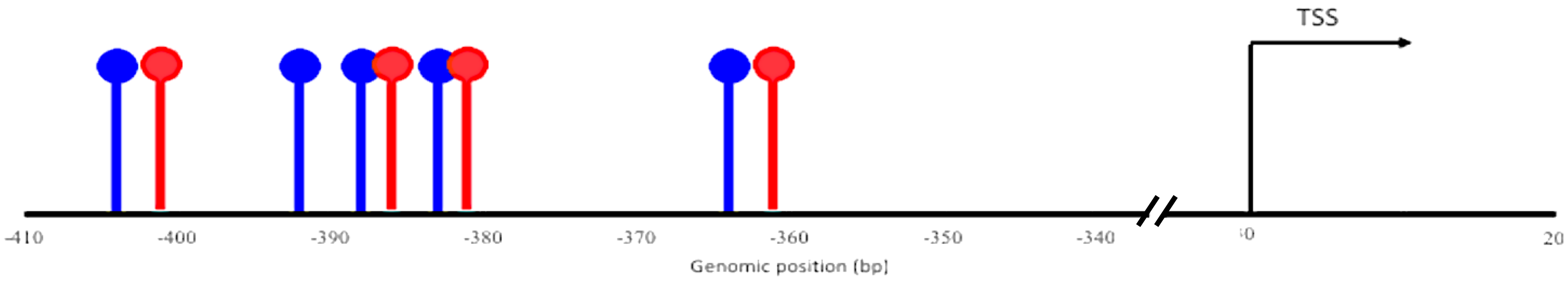

Figure S1D. A schematic illustration of the assayed CpG sites in the *PER2* gene in the study. A total of 9 CpG sites were assayed in the *PER2* gene. Red represents the CpG sites associated with glucose metabolism (raw P ≤ 0.1). TSS: transcription start site.

***PER3* gene**

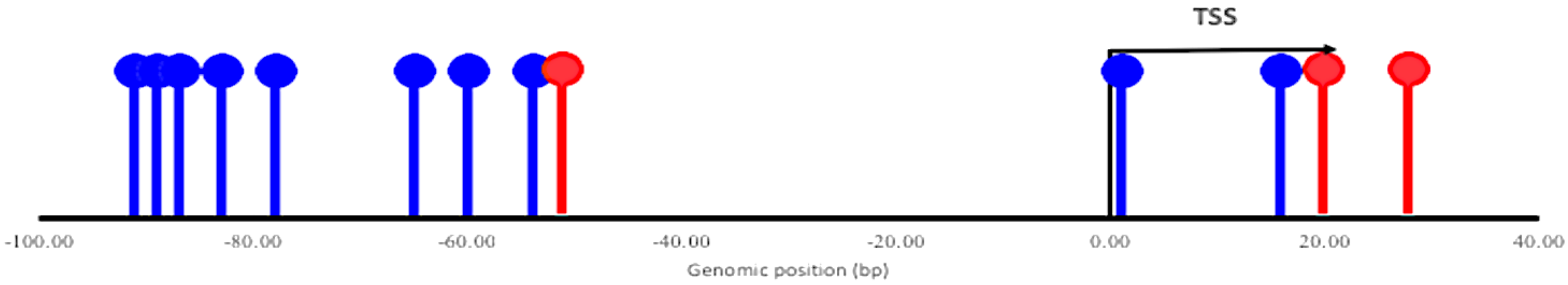

Figure S1E. A schematic illustration of the assayed CpG sites in the *PER3* gene in the study. A total of 13 CpG sites were assayed in the *PER3* gene. Red represents the CpG sites associated with glucose metabolism (raw P ≤ 0.1). TSS: transcription start site.
